# Influence of Fission Product Distribution in Medium-Burnup UO_2_ Fuel on Cracking Behavior

**DOI:** 10.3390/ma18153571

**Published:** 2025-07-30

**Authors:** Dongsheng Xie, Chuanbao Tang, Tong Fu, Jiaxuan Si, Changqing Teng, Lu Wu

**Affiliations:** 1 The First Sub-Institute, Nuclear Power Institute of China, Chengdu 610213, China; xds24240808@163.com (D.X.); futong_97@163.com (T.F.); sijiaxuan9610@126.com (J.S.); 15928669112@163.com (C.T.); 2 State Key Laboratory of Advanced Nuclear Energy Technology, Nuclear Power Institute of China, Chengdu 610213, China; tangcb_1@sina.cn; 3 Science and Technology on Reactor System Design Technology Laboratory, Nuclear Power Institute of China, Chengdu 610213, China

**Keywords:** nuclear fuel, fission products, bubbles, grain boundary cracking, intragranular cracks

## Abstract

This investigation employs focused ion beam (FIB) and transmission electron microscopy (TEM) techniques to systematically analyze the distribution characteristics of fission products in medium-burnup (40.6 GWd/tU) UO_2_ fuel and their impact on fuel cracking behavior. The findings indicate that grain boundary embrittlement is predominantly attributed to the accumulation of spherical particles of solid fission products, including Mo, Ru, Rh, and Pd, which preferentially segregate around impurity particles, leading to localized stress concentration. Intragranular cracks are associated with the strip-like segregation of fission elements and the amorphization process. It also reveals that the size and number density of intragranular Xe bubbles are ~6.24 ± 0.24 nm and 5.2 × 10^22^ m^−3^, respectively, while Xe did not, under the analyzed conditions, significantly influence crack nucleation. This research elucidates the correlation mechanism between fission product distribution and fuel cracking behavior at medium burn up, offering experimental evidence to enhance the reliability and safety of nuclear fuel assemblies.

## 1. Introduction

Nuclear fuel assemblies serve as the pivotal energy conversion medium in nuclear energy systems, with their performance directly influencing the neutron economy, thermal–hydraulic properties, and accident tolerance capabilities of the reactor. Consequently, investigations into the behavior of fission products are of paramount importance for the design, operation, and safety evaluation of nuclear fuel assemblies [1,2,3]. The accumulation of fission products results in an increase in the burnup of nuclear fuel, thereby impacting the reactivity and power output of the fuel [4,5,6,7,8]. Through the analysis of fission products, one can accurately evaluate the burnup level of nuclear fuel, offering essential data for the operation and management of nuclear reactors. Conducting research on the effects of fission products on the microstructural morphology and structural integrity of fuel can lead to significant improvements in the reliability of nuclear fuel assemblies [9].

During the operational stage of a nuclear reactor, for every three to four fission events in UO_2_ fuel, one inert gas atom is produced. Approximately 15% of the generated fission products consist of inert gases such as Xe and Kr, with Xe being produced at a rate seven times higher than that of Kr. These inert gases exhibit extremely low solubility in the UO_2_ matrix and are present in the form of nanoscale inclusions or bubbles within and between the grains of the fuel matrix, or they may escape from the matrix. When the local burnup of the fuel pellet exceeds 50 GWd/tU and the local irradiation temperature remains below 1100 °C, the fuel pellet undergoes a structural transformation into a high-burnup structure (HBS) [10]. This transformation is accompanied by grain refinement, leading to the formation of a distinct microstructural morphology. Within the HBS region, a significant number of Xe atoms diffuse into the bubbles, potentially generating bubbles with pressures exceeding equilibrium levels. These high-pressure Xe bubbles can induce localized high stresses, which may be responsible for fuel cracking and fragmentation. Under accident conditions, this can have severe implications for the safety of nuclear installations [11,12,13,14,15,16]. Beyond fission gas, the metallic characteristics of solid fission products, such as Mo, Ru, Rh, Pd, and Tc, also possess a substantial fission yield, and their precipitation may also lead to pronounced disparities in local thermal conductivity and mechanical properties. Notably, the fuel fragmentation phenomenon is not merely confined to the overpressure condition of bubbles at grain boundaries of the HBS region. During the service life of the fuel, creep-induced grain boundary cracking can also give rise to fractures [17]. More significantly, under low-temperature conditions, the thermal spike effect induced by fission fragments will exert a notable influence on the creep behavior of the fuel, that is, fission-induced creep [18]. Consequently, prior to the formation of HBS at the pellet edges, even though fission products remain at the nanoscale level, the morphology and distribution of different types of fission products can, to a certain degree, also trigger fuel cracking. This, in turn, can significantly impact the safety of the reactor under reactivity-initiated accident (RIA) conditions. However, as out-pile creep tests are unable to effectively capture the true fission-induced creep behavior and the causes of fuel cracking, the characterization of the microstructure under medium-burnup conditions represents an effective approach to evaluating the early crack behavior of fuel. At this stage, the distribution of fission products is sufficient to influence the structure and properties of fuel pellets in local regions, especially grain boundaries. Nevertheless, owing to the complexity associated with the microscopic characterization of relevant materials, research on the influence of fission products on fuel crack behavior prior to the formation of HBS remains relatively scarce.

Consequently, this study, a combination of focused ion beam (FIB) and transmission electron microscope (TEM) techniques was utilized to explore the distribution characteristics of the nanoscale intergranular and intragranular fission products in UO_2_ samples with a medium burnup of 40.6 GWd/tU, and we further analyzed the potential impact of the fission product structure on fuel cracking behavior. This research endeavor is anticipated to contribute significantly to enhancing the reliability, safety, and economic viability of nuclear fuel assemblies, thereby fostering the sustainable development of nuclear energy.

## 2. Irradiation History and Characterization Methods

In this study, UO_2_ fuel specimens with a diameter of 8.1 mm were irradiated in a High Flux Engineering Testing Reactor (HFETR) at the Nuclear Power Institute of China (NPIC) for 960 full power days. The average linear heat generation rate was 18.3 kW/m, and the temperature at the edge of the fuel pellet in this study ranged from 300 to 400 °C during the irradiation period, as monitored by the thermocouples attached to the outer surface of the specimen. The post-irradiation examinations were performed in the hot cells of the NPIC. For the burnup measurement, Nd was employed as the burnup monitoring entity. The contents of the fission products ^145^Nd and ^146^Nd were determined via isotope dilution mass spectrometry. Based on these results, the amount of uranium that participated in fission was deduced, and then the burnup value was calculated. Single-point burnup measurements were carried out on three points in the fuel’s edge region, with the average burnup value of 40.6 GWd/tU.

Before microstructural characterization, the post-irradiated sample was sealed within epoxy resin and then was abraded using SiC paper of up to 4000 mesh, followed by mechanical polishing with 6, 3, 1, 0.25, and 0.1 μm water-free diamond suspensions. At last, the sample was sprayed with gold for electric conduction. The limited final sample size and consequently reduced radioactivity levels enabled the safe removal of the fuel slice from the hot cell and its transfer to a radiologically controlled laboratory equipped with advanced characterization instruments. A scanning electron microscope (SEM) was applied to examine the macroscopic morphology of UO_2_ pellets. Subsequently, the cross-sectional TEM samples were prepared by the FIB lift-out technique (ThermoFisher Helios 5 CX, Waltham, MA, USA), to make them suitable for high-resolution transmission characterization. A 1 μm thick protective layer of platinum was deposited by FIB on the regions of interest before lift-out. FIB shaping of the specimens was performed at 30 kV ion beam voltage with a final milling step at 2 kV in order to control Ga implantation. The structure and elemental composition of fission products in different characteristic regions were analyzed through a scanning transmission electron microscope (STEM) and energy dispersive spectroscopy (EDS) using an aberration-corrected transmission electron microscope (ThermoFisher Spectra-300, Waltham, MA, USA), with the beam acceleration voltage, current, and EDS acquisition time of 300 KV, 30–100 pA, and 15 min, respectively. For the purpose of comparison, the unirradiated UO_2_ samples were characterized via the same sample preparation methodology. Furthermore, three irradiated TEM samples were subjected to testing to rule out the uncertainties associated with the phenomena. The experimental findings were analyzed solely on the basis of results that were demonstrably reproducible.

## 3. Results and Discussion

**Figure 1:**
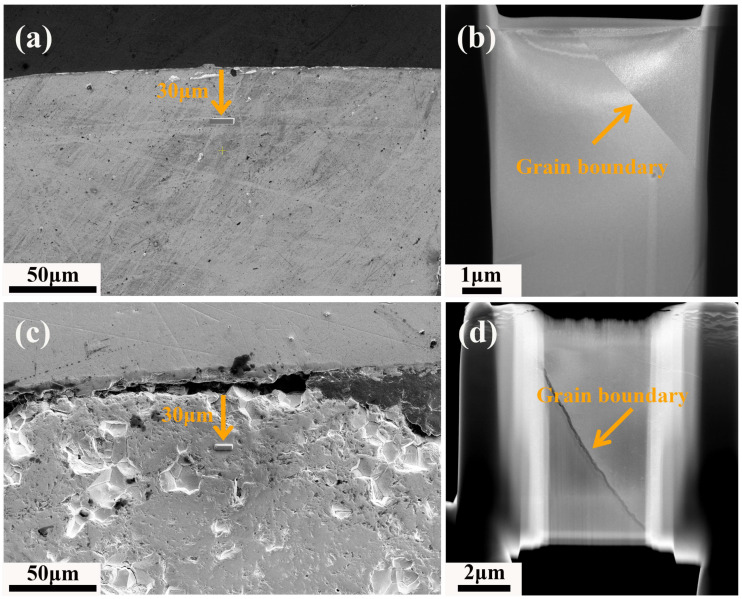
Schematic illustration of the FIB sampling process and the overall morphology of the TEM sample: (**a**,**b**) unirradiated UO_2_ sample, (**c**,**d**) irradiated UO_2_ sample with a burnup of 40.6 GWd/tU. (**a**) SEM image with a planar surface, (**b**) STEM-BF image with a planar grain boundary, (**c**) SEM image with grain detachment at the surface, (**d**) STEM-BF image with a cracked grain boundary.

Figure 1 presents the SEM images of the pellet edge morphologies of the unirradiated sample and irradiated sample, along with the STEM bright field (STEM-BF) images of the grain boundary states in the samples prepared by FIB. Due to the self-shielding effect, the burnup at the pellet edge is typically significantly higher than at the center [10], and thus to investigate the region with higher burnup within this sample, the sampling location was chosen at a distance of 30 μm from the pellet edge, corresponding to a radial position ratio of r/r0 > 0.99. For the unirradiated samples, the edges of the pellets exhibit a smooth surface morphology (Figure 1a), and the corresponding STEM-BF images reveal that the grain boundary region are intactly bonded (Figure 1b). In contrast, as observed from the morphological image in Figure 1c, a notable grain detachment phenomenon is present, while the grain size remains relatively uniform, with no formation of a high-burnup structure, and the STEM-BF image presented in Figure 1d reveals the cracking phenomenon at the grain boundaries more conspicuously, which suggests that when the burnup attains 40.6 GWd/tU, under the influence of fission-induced creep behavior, the grain boundary cohesion is significantly reduced. Subsequently, a detailed analysis was carried out on the sample with the burnup of 40.6 GWd/tU.

**Figure 2:**
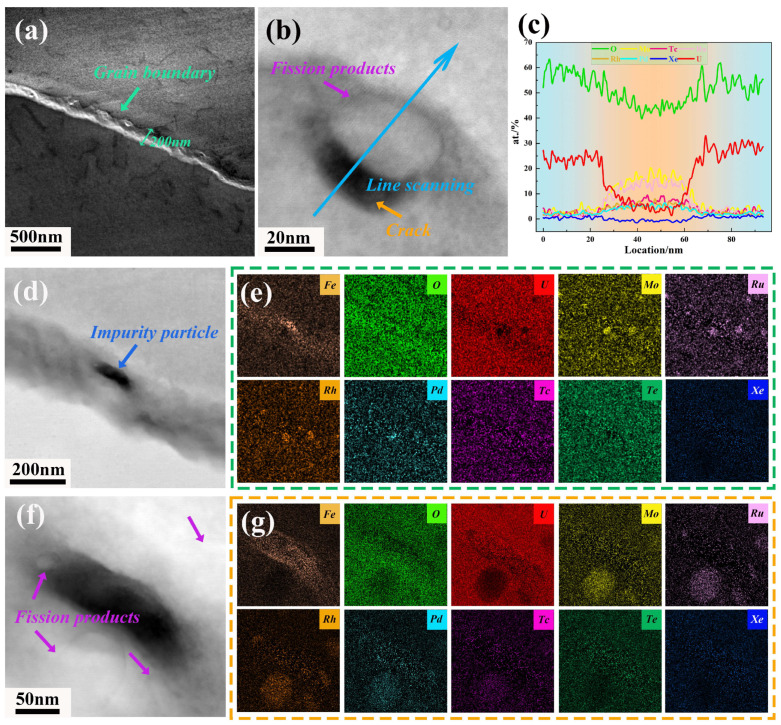
STEM images of the grain boundary feature in irradiated UO_2_ fuel (40.6 GWd/tU). (**a**) STEM-DF image illustrating the characteristics of the grain boundary region and the distribution of dislocations around grain boundary; (**b**) fission products at the grain boundary; (**c**) EDS line scanning results according to the blue arrow in (**b**); (**d**,**e**) distribution of fission products at the grain boundary region and the corresponding EDS mapping results; (**f**,**g**) Enrichment of fission products in the vicinity of impurity particles and the corresponding EDS mapping results.

Figure 2 provides a further analysis of the microscopic characteristics at the grain boundaries by TEM. The distribution of fission elements near the grain boundary was characterized by EDS to compare the types, sizes, and morphologies of fission products in the grain interior versus at the grain boundary. The morphological features of the grain boundary region differ from those of conventional materials, with an extended irradiation defect influence zone of approximately 200 nm, and a high density of dislocations could be observed, distributed on both sides of the grain boundary (Figure 2a). During neutron irradiation, high-energy cascade collisions form local thermal peaks near the grain boundary, intensifying the migration and recombination of defects [19]. Moreover, the temperature at the edge of the fuel pellet is relatively low, and the static capture effect of the grain boundary on defects in this area is more pronounced, resulting in a higher vacancy concentration. In Figure 2d,e, it can be seen that fission elements tend to form spherical particles at the grain boundary with the enrichment of Mo, Ru, Rh, and Pd under the diffusion effect of the temperature gradient and pipe diffusion at dislocations [20]. Regarding the segregation of fission elements, studies have shown that Ru has a strong segregation tendency at the a0/2<110>{110} edge dislocation core in UO_2_ [21], while Mo can strengthen the U-O bonding across the grain boundary in UO_2_, thereby enhancing the cohesion of grain boundary [22]. However, in the context of the fission-induced creep process, the formation of precipitates/particles at grain boundaries can reduce lattice match at the grain boundary, thus causing local stress concentration and triggering the accumulation of dislocations in the vicinity of grain boundaries and thus leading to grain boundary cracking, which is common in superalloys [23,24]. In Figure 2b, the formation of a crack is observable at the edge of the particles, with the crack width of ~15 nm, and the corresponding EDS line scanning result (Figure 2c) reveals a notable depletion of the U and O elements of the fission product, which is predominantly enriched with Mo and Ru elements, with the content of 20.4 at.% and 16.1 at.% (Figure 2c), respectively. Meanwhile, trace amounts of Rh and Pd are also present.

Generally, there is a significant driving force for gas atoms to segregate to extended defects such as grain boundaries and dislocations, subsequently nucleating to form bubbles at these “sinks”. After segregating to the grain boundary, the fission gas may be released to the fuel gas cavity through the rapid diffusion of individual gas atoms along the grain boundary or through the permeation network formed by interconnected bubbles [25]. However, no bubble enrichment was found at the grain boundary in this study, which is typically related to the diffusion behavior under different temperature conditions. The diffusion coefficients of Xe, U, and O in UO_2_ are related to both temperature and the proportion of Xe. With the increase in the proportion of Xe, the number, size, and gyration radius of Xe clusters increase significantly. At 10% Xe, the bubble nucleation phenomenon is quite pronounced [26], which suggests that when the burnup does not reach a certain value, the nucleation ability of Xe bubbles at the grain boundary is not prominent. Figure 2f,g show the microscopic characteristics near impurity particles at the grain boundary. It can be observed that spherical fission product particles are highly concentrated around the impurity particles. Due to the high mismatch between the crystal structure of Fe and UO_2_, lattice distortion is formed around the particles, generating local strain, which attracts the preferential segregation of fission elements in this area. Therefore, it can be concluded that the high vacancy concentration, impurity particles, and fission product particles distributed in the grain boundary region may collectively contribute to the grain boundary embrittlement of the fuel, leading to grain boundary cracking during service and impacting the structural integrity of the fuel.

**Figure 3:**
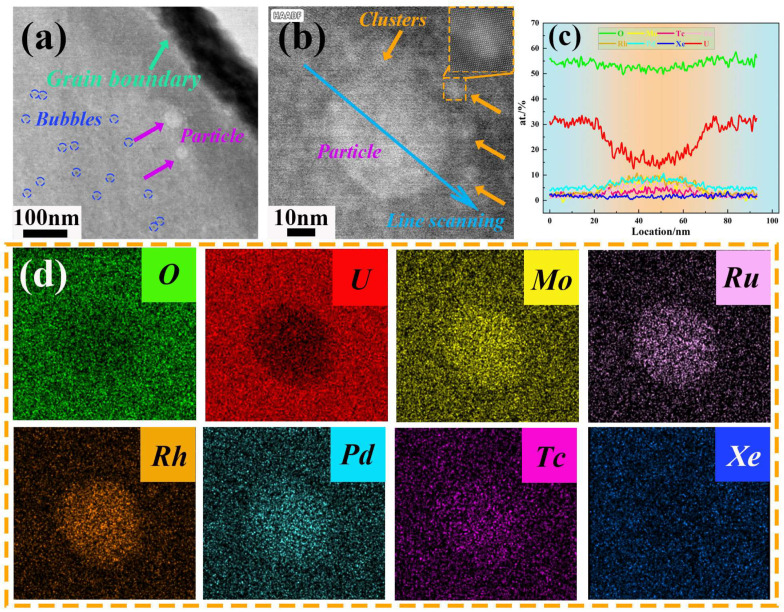
Distribution of fission products in the vicinity of grain boundaries in irradiated UO_2_ fuel. (**a**) HAADF-STEM image; (**b**) high-resolution image of the ellipsoidal particle and clusters in (**a**); (**c**) EDS line scanning results according to the blue arrow in (**b**); (**d**) EDS mapping results of the ellipsoidal particles.

Subsequently, an analysis was conducted on the fission products located approximately 500 nm away from the grain boundary, as depicted in Figure 3. The findings revealed that at the ~100 nm region, only a limited number of ellipsoidal particles had formed, with the largest reaching a size of approximately 50 nm (Figure 3a). Adjacent to the particle, several nanoclusters with the size of ~5 nm can be observed in Figure 3b, and the corresponding atomic HAADF-STEM image in the top right corner of Figure 3b confirms that the atomic arrangement within the cluster is consistent with the matrix, ruling out the presence of the commonly reported Hexagonal Close-Packed structure precipitation [27]. From the corresponding EDS line scanning and mapping results in Figure 3c,d, it is evident that the O content of this particle exhibits only a slight reduction, which is distinct from the fission product particles at grain boundaries, while the U content has significantly decreased to 12.5 at.%. Moreover, this particle is also enriched in Mo, Ru, Rh, Pd, and Tc, with their respective contents reaching ~9.8 at.%, ~9.2 at.%, ~9.9 at.%, ~10.6 at.%, and ~5.3 at.%. In contrast, within the region ranging from 100 to 500 nm away from the grain boundary, the formation of such types of particles and clusters is scarcely observable. In addition, regarding Xe bubbles, they begin to emerge at a distance of 200 nm from the grain boundaries, forming a “Xe bubble-free zone” in the vicinity of the grain boundaries, with a complete absence of bubbles around the grain boundary, which is attributed to the grain boundary sink effect [28], indicating that grain boundary cracks serve as an escape pathway for fission gas [29].

**Figure 4:**
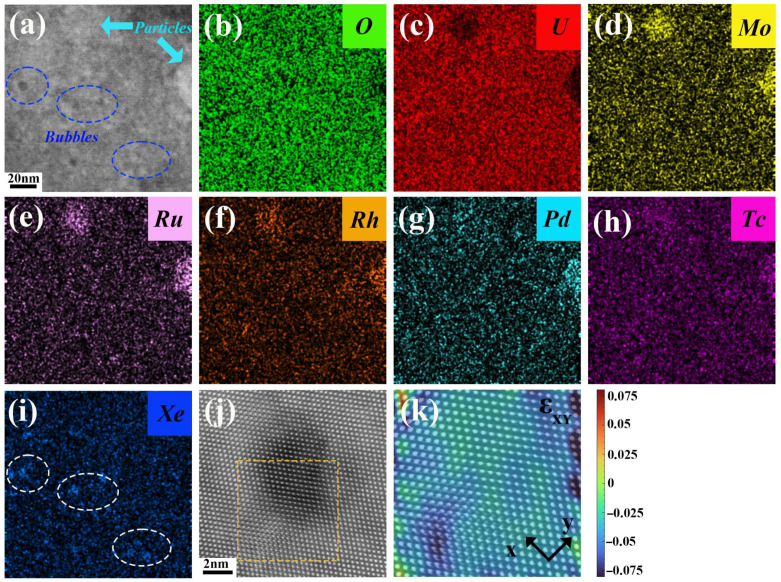
Bubble distribution near the grain boundaries of the irradiated UO_2_ fuel. (**a**) HAADF-STEM image; (**b**–**i**) EDS mapping results of (**a**); (**j**) atomic arrangement near the bubble; (**k**) GPA of the area within the dashed line in (**j**).

Figure 4 further illustrates the distribution characteristics of various types of fission products (bubbles and particles) in the vicinity of grain boundaries. The EDS results reveal that there is no interaction between Xe bubbles and non-volatile fission elements such as Mo, Ru, Rh, Pd, and Tc (Figure 4b–i). The bubble sizes are relatively uniform, approximately 5 nm, and they typically nucleate at the edges of dislocations (Figure 4j). Generalized plane strain analysis (GPA) indicates that a local compressive stress field is generated in the dislocation region (Figure 4k), which facilitates the segregation of Xe atoms near defects and their gradual growth into Xe bubbles. Molecular dynamics simulations indicate that Xe has a tendency to segregate preferentially at dislocations, thus inducing the nucleation of Xe bubbles [30]. Moreover, during the growth of these bubbles, the high stresses within and around the Xe bubbles are locally relieved via dislocation lines or dislocation loops. Consequently, dislocations play a crucial role in both the nucleation and growth processes of the bubbles [31].

**Figure 5:**
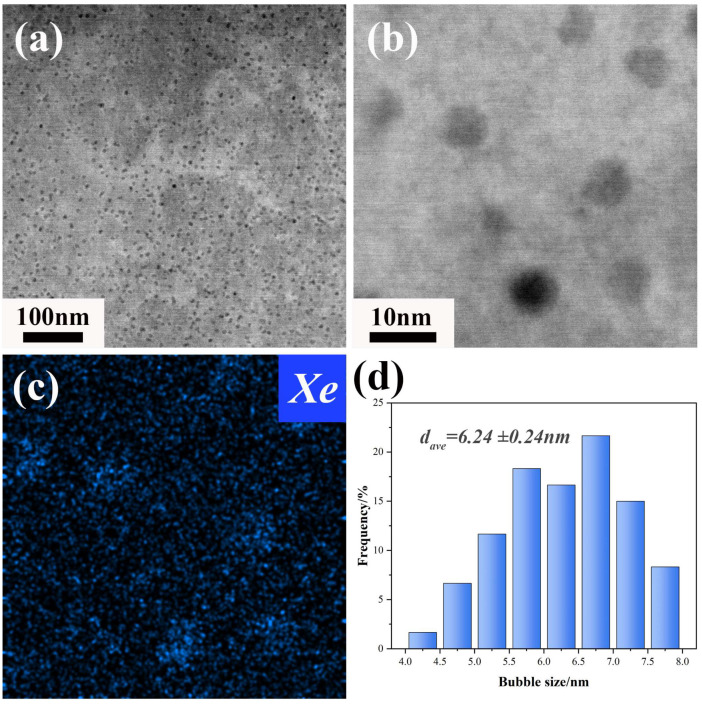
Intragranular bubble distribution of irradiated UO_2_ fuel. (**a**) HAADF-STEM image of the overall distribution; (**b**) high-resolution image of the bubbles; (**c**) Xe EDS mapping results of (**b**); (**d**) the statistical distribution plot of bubble sizes in (**a**).

Figure 5 presents the characterization and statistical analysis of the characteristics of Xe bubbles over a wider range within the grains. Figure 5b,c reveal that the Xe bubbles are distributed relatively uniformly, with an average size of approximately ~6.24 ± 0.24 nm (Figure 5d) with the bubble sample size of 1604 in Figure 5a. By means of the convergent beam electron diffraction, the thickness of the sample was measured to be ~83 nm. Consequently, the number density of Xe bubbles within the grains in Figure 5a reached 5.2 × 10^22^ m^−3^. In a previous study by S. Kashibe [32], the sizes and number densities of Xe bubbles under conditions of 44 GWd/tU and 83 GWd/tU were found to be ~3.9 nm and 7 × 10^23^ m^−3^, and ~4.7 nm and 4 × 10^23^ m^−3^, respectively. In contrast, the bubble size in the present study is slightly larger, while the number density is relatively lower. The factors contributing to these subtle differences are associated with temperature and uranium enrichment. Findings from molecular dynamics studies indicate that the pressure of Xe nanobubbles decreases with increasing temperature and bubble size [33]. Therefore, when the Xe content remains constant, temperature and bubble size interact to maintain pressure equilibrium. It should be noted that the size and distribution of bubbles are also related to the interaction between fission products and irradiation-induced defects [34], as well as the re-dissolution of fission gas [35], and thus further research in this area is required in the future. Under high-burnup conditions, influenced by grain refinement, bubbles typically exhibit complex morphologies. In HBS, the bubbles between sub-grains initially emerge as tiny, closely packed lenticular shapes, resembling typical intergranular lenticular bubbles. As the burnup level deepens, the bubbles gradually grow in size and thickness and interconnect in local regions, forming larger and more intricate bubbles that can span multiple low-angle grain boundaries, thus facilitating the release of fission gases [36]. However, in this study, as the HBS has not yet developed, Xe bubbles predominantly exist in a stable state within the grains and exert negligible influence on the cracking behavior of the fuel.

**Figure 6:**
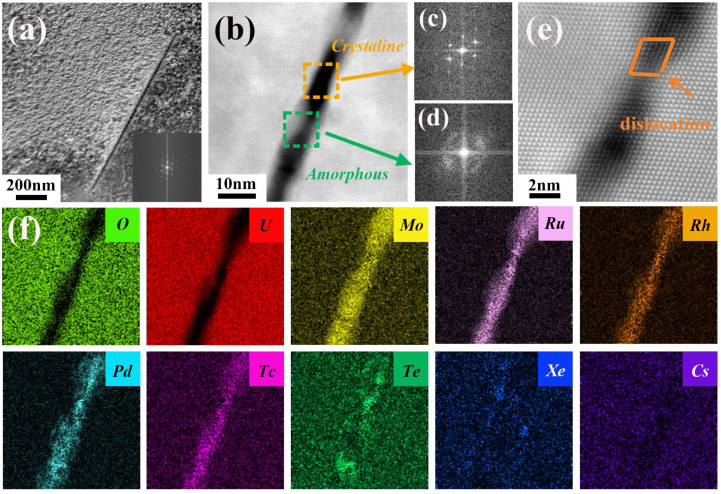
Intragranular striped cracks in UO_2_. (**a**) STEM-DF image and the corresponding SAED pattern; (**b**) HAADF-STEM image of the striated crack region; (**c**,**d**) SAED patterns corresponding to the area shown in (**b**); (**e**) atomic arrangement distribution within the striped region; (**f**) EDS mapping results of (**b**).

Figure 6a depicts the microstructure of the strip feature in the matrix of irradiated UO_2_. To ascertain whether this feature represents a crack or a fission product, a detailed analysis was conducted using SAED and EDS. The analysis reveals that it has a width of approximately 10 nm, primarily constituted by the segregation of Mo, Ru, Rh, Pd, and Tc elements, with an extra enrichment of Te elements (Figure 6b,f). This finding is corroborated by Kessler et al. [37], who, through TEM and DFT, confirmed the presence of Pd-rich telluride phases in UO_2_ fuel, which indicates a strong binding affinity between Pd and Te. Furthermore, the enrichment of Xe elements was observed at the boundaries of the strip, and HAADF-STEM revealed the formation of dislocations within the region (Figure 6e). During neutron irradiation, the recoil effect generated during nuclear fission causes lattice damage, leading to the formation of initial irradiation damage crack traces [38], and simultaneously, dislocations are produced within these traces. Fission elements have a tendency to segregate at the lattice distortion and dislocation core regions that they create [21]. Under the influence of high temperatures, the recovery process results in the shortening or disappearance of the crack traces, and the damage effect caused by fission gradually diminishes. However, due to the uncontrollable nature of this process, only certain orientations leave behind a strip ~1 mm in length. In conjunction with the SAED obtained from within the region, it is evident that a partial amorphization has occurred (Figure 6c,d), leading to the formation of a high-energy distortion zone. Typically, as the local concentration of point defects keeps rising, a certain critical value will be reached, and subsequently the lattice becomes unstable and collapses to form an amorphous structure [32]. The segregation of fission elements in the high-energy fission recoil region preferentially acts as a defect sink, effectively absorbing the point defects generated in the surrounding regions, and thus an amorphous structure is formed in the local area. The phenomenon of irradiation-induced local amorphization can also occur in FeCrAl [39] and Zr alloys [40,41], which is generally the combined result of irradiation dose and temperature. Overall, this area, which has undergone the sequence of fission track formation, fission element segregation, and amorphization, will result in a modulus mismatch within the local region and gradually evolve into cracks within the matrix. This transformation is substantiated by the contrast image obtained from HAADF-STEM, providing a clear visualization of the microstructural changes.

## 4. Conclusions and Prospects

This study, through the integration of FIB and TEM techniques, has systematically analyzed the distribution characteristics of fission products in medium-burnup UO_2_ fuel and their impact on fuel cracking behavior. The following key conclusions have been drawn:

(1) Grain boundary embrittlement and the formation of intragranular striped cracks are the primary mechanisms of fuel cracking at medium burnup, which are closely associated with the segregation of fission products and localized stress concentration.
(2) In the grain boundary region, solid fission products, including Mo, Ru, Rh, and Pd, are enriched in spherical particles and tend to form high-density segregation around impurity particles, leading to localized stress concentration and grain boundary embrittlement.
(3) The formation of an intragranular striped area is closely related to the segregation of fission elements and the amorphization process, which gradually transform into intragranular cracks under irradiation conditions.
(4) Under 40.6GWd/tU burnup conditions, the size and number density of intragranular Xe bubbles are ~6.24 ± 0.24 nm and 5.2 × 10^22^ m^−3^, respectively, and Xe did not exert a significant influence on crack nucleation under the conditions analyzed in this study. Instead, a “Xe bubble free zone” in the vicinity of the grain boundaries was formed, indicating that grain boundary cracks serve as an escape pathway for fission gas.

Based on the findings of this study, future research could be conducted in several directions to enhance the reliability, safety, and economy of nuclear fuel assemblies:

(1) Additional experimental studies should be conducted, complemented by atomic-scale simulations and molecular dynamics simulations, to deeply elucidate the nucleation, growth, and migration mechanisms of fission products in UO_2_ fuel, particularly focusing on the dynamic behavior of Xe gas bubbles and solid fission product clusters with irradiation defects.
(2) For UO_2_ fuel under different burnup conditions, the distribution characteristics of fission products and their impact on fuel performance should be investigated in more detail, exploring the critical conditions for fuel swelling, cracking, and gas release at different burnup levels.
(3) In view of the fact that cracks emerge in the fuel at medium-burnup levels, during the fabrication of fuel pellets, it is necessary to explore the possibility of enhancing the stability of fission products within the matrix to a certain extent through doping while precisely controlling the impurity content. This would effectively impede the migration of fission elements towards the grain boundaries, thereby enhancing the stability of the microstructure.
(4) During the operation of the nuclear reactor, stringent control over power and temperature management is of the utmost importance and is essential for ensuring the safety performance of fuel pellets throughout every stage of the production and utilization process.

Through the in-depth exploration of these research directions, the performance of nuclear fuel assemblies can be significantly improved, contributing to the sustainable development of nuclear energy technology and providing essential support in producing a clean, safe, and efficient energy system.

## Data Availability

The original contributions presented in this study are included in the article. Further inquiries can be directed to the corresponding author.
